# Solid index versus intraoral scanners in the full-arch implant impression: in vitro trueness evaluation

**DOI:** 10.1186/s13104-020-05353-2

**Published:** 2020-11-03

**Authors:** Francesco Guido Mangano, Matteo Bonacina, Federico Mandelli, Fabio Marchiori

**Affiliations:** 1 Department of Prevention and Communal Dentistry, Sechenov First State Medical University, Moscow, Russia; 2 Ars and Technology, Sotto Il Monte Giovanni XIII, Bergamo, Italy; 3 Private Practice, Milan, Italy; 4 Santa Maria Di Sala, Venti 07, Venezia, Italy

**Keywords:** Solid index, Intraoral scanners, Full arch implant impression, Trueness

## Abstract

**Objectives:**

To assess the trueness of a solid index (SI) in the full-arch (FA) implant impression, and to compare it with that of two intraoral scanners (IOSs). A type-IV gypsum model of a completely edentulous patient with 8 implant scanbodies (SBs) was scanned with a desktop scanner (7Series®) to obtain a reference virtual model (RVM), and with two IOSs (CS 3700® and Emerald S®). Five scans were taken with each IOS. Based on the RVM, an SI (custom tray consisting of hollow cylinders connected by a bar) was fabricated and used to capture a physical impression of the model; from this, a second gypsum model was derived and scanned with a desktop scanner (D15®). The SI-derived and the IOSs-derived models were superimposed onto the RVM, to evaluate trueness.

**Results:**

The overall mean trueness was 29 μm (± 26) for the SI-derived model, versus 42.4 μm (± 14.7) for CS 3700® and 52.2 μm (± 4.6) for Emerald S®. Despite its limitations (in vitro design, a limited number of models evaluated, RVM captured with a desktop scanner) this study supports the use of SI for FA implant impressions. Further studies are needed to confirm this evidence.

## Introduction

Several studies [1–3] and systematic reviews [4, 5] have reported that intraoral scanners (IOS) are not sufficiently accurate to capture impressions in completely edentulous patients, which can be used for the fabrication of full-arch (FA) implant-supported restorations via a fully digital workflow.

This inaccuracy seems to depend intrinsically on the IOS, and on the mechanism by which it ‘attaches’ to each other the individual images or frames captured during the scan [6]. The error grows with the stitching of the images and with the progression of the scan [6]. Additional factors that can determine inaccuracy depend on the operator (scanning strategy) [7], patient (number, position, inclination and depth of the implants) [8], environmental conditions (light) and transfer of the implant position, the so-called scanbody (SB) [9, 10]. The design and materials with which SBs are made, together with manufacturing tolerances, have been documented to cause errors [9, 10]. The congruence between SBs mesh and implant library files can also play a role in determining errors in implant position within the prosthetic computer-aided design (CAD) software [11].

Schmidt et al. [12] used a customised solid index (SI), three-dimensionally (3D) designed and fabricated to capture with little material a high-quality physical impression that records the distance between implants with minimal error. This SI can be used to pour a physical model that is scanned with a desktop scanner, and on which the dental technician can model an accurate prosthetic superstructure within a digital workflow [12]. In details, the authors prepared a reference model of a partially edentulous maxilla with four implant analogues in the posterior sectors, they screwed in the SBs and scanned it with an industrial reference scanner [12]. The authors scanned the same reference model 10 times with an IOS (Trios 3, 3-Shape, Copenhagen, Denmark) [12]. They then recorded an impression with the SI that consisted of four hollow connected cylinders with a parallelepiped of known dimensions positioned on the palate [12]. A minimal amount of polyether was used to solidarise the SBs to the SI. After hardening, the SBs were unscrewed and the SI was sent to the laboratory, where it was measured with a coordinate measuring machine (CMM) for assessing the distances between the implants [12]. Finally, 10 conventional analogue impressions of the reference model were captured with classic trays and materials, from which the technician poured plaster casts which were, in turn, probed with a CMM [12]. The authors compared the accuracy of the different methods and found that significantly higher trueness was achieved with the SI [12]. These results were confirmed in vivo, in a series of three cases successfully completed using the aforementioned SI protocol [12].

In another in vivo study, Mandelli et al. [13] developed this concept. They presented the clinical results obtained with the solid index impression protocol (SIIP), a technique that uses an SI (a custom tray consisting of hollow cylinders connected by a bar) to capture accurate impressions of multiple implants for the fabrication of implant-supported fixed FAs. In this study, direct intraoral digital impressions (True Definition®, 3 M ESPE, Maplewood, MN, USA) were obtained from 5 fully edentulous patients treated with 4 implants. In addition, a physical impression was taken with the SI for the fabrication of an FA implant-supported prosthesis [13]. The index, linked to the implant transfers, was sent to the laboratory and used to pour an SI-derived plaster cast; this SI-derived cast was scanned with a desktop scanner for the fabrication of the final prosthesis [13]. In all cases, the SIIP provided excellent accuracy, and the FA restorations obtained from the SI-derived model demonstrated optimal clinical precision [13]. Marked differences in accuracy were found between the virtual models derived from SIIP and the ones derived from intraoral scans [13]. The authors concluded that the SIIP technique is clinically reliable for the FA implant impressions, and more accurate than the direct intraoral impression using IOS [13].

However, it is known from several in vitro studies that different IOSs demonstrate statistically different levels of accuracy [1, 2, 6, 14, 15].

The purpose of this in vitro study was therefore to verify the reliability and trueness of the SI in the FA implant impressions, and to compare the trueness of an SI-derived virtual model with those of models captured with two IOSs which have never been tested in this context.

## Main text

**Fig. 1:**
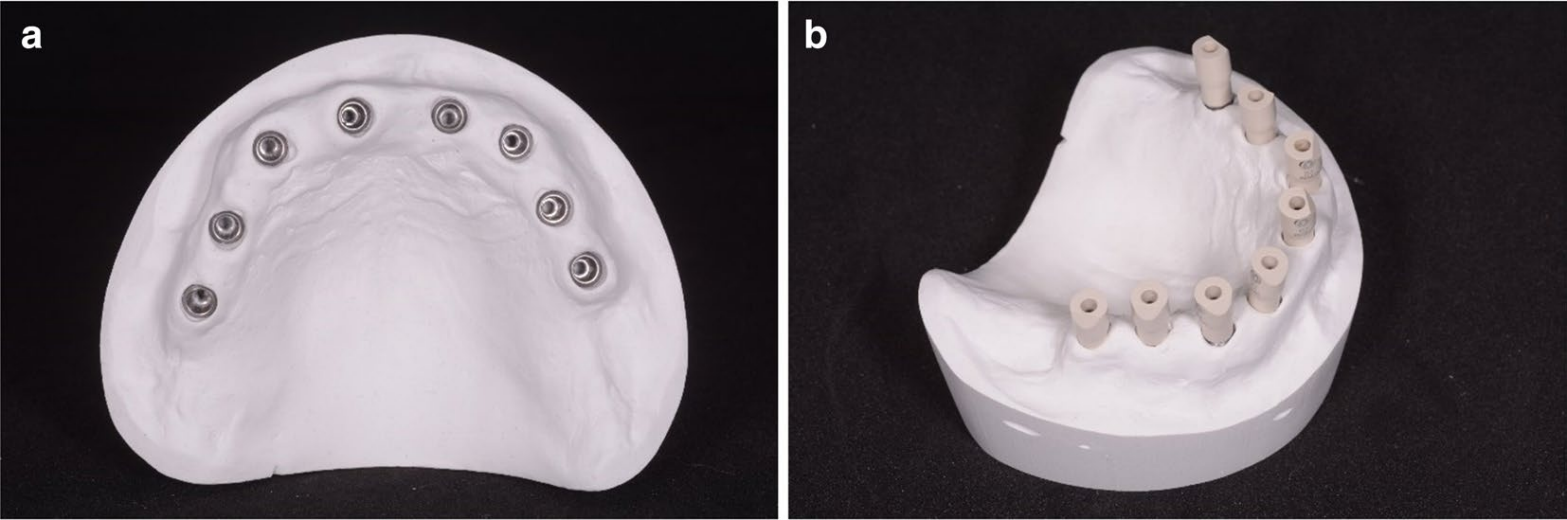
The reference stone cast model. **a** The reference model with 8 implants analogs (BT Safe KR®) and **b** with the proprietary SBs screwed on

A reference type-4 gypsum model of a completely edentulous maxilla was prepared with 8 implant analogues (BT Safe KR®, Biotec Srl, Dueville, Vicenza, Italy) (Fig. 1a) and the related SBs screwed onto them (Fig. 1b).

This model was scanned with two IOSs (CS 3700®, Carestream Dental, Atlanta, GA, USA; and Emerald S®, Planmeca, Helsinki, Finland). Each IOS captured five scans (meshes). The operator was a dentist expert in intraoral scanning who used a zigzag scanning strategy [2, 11], starting from the right posterior area, then moving mesially until the incisal area and finally distally, to the left posterior area. The scans were captured with the latest available versions of the IOSs software in August 2020, in a room with the same artificial light and temperature (22 °C). The intraoral scans were cut and trimmed using a pre-formed template for uniformity, and saved in stereolithographic (STL) format for analysis.

**Fig. 2:**
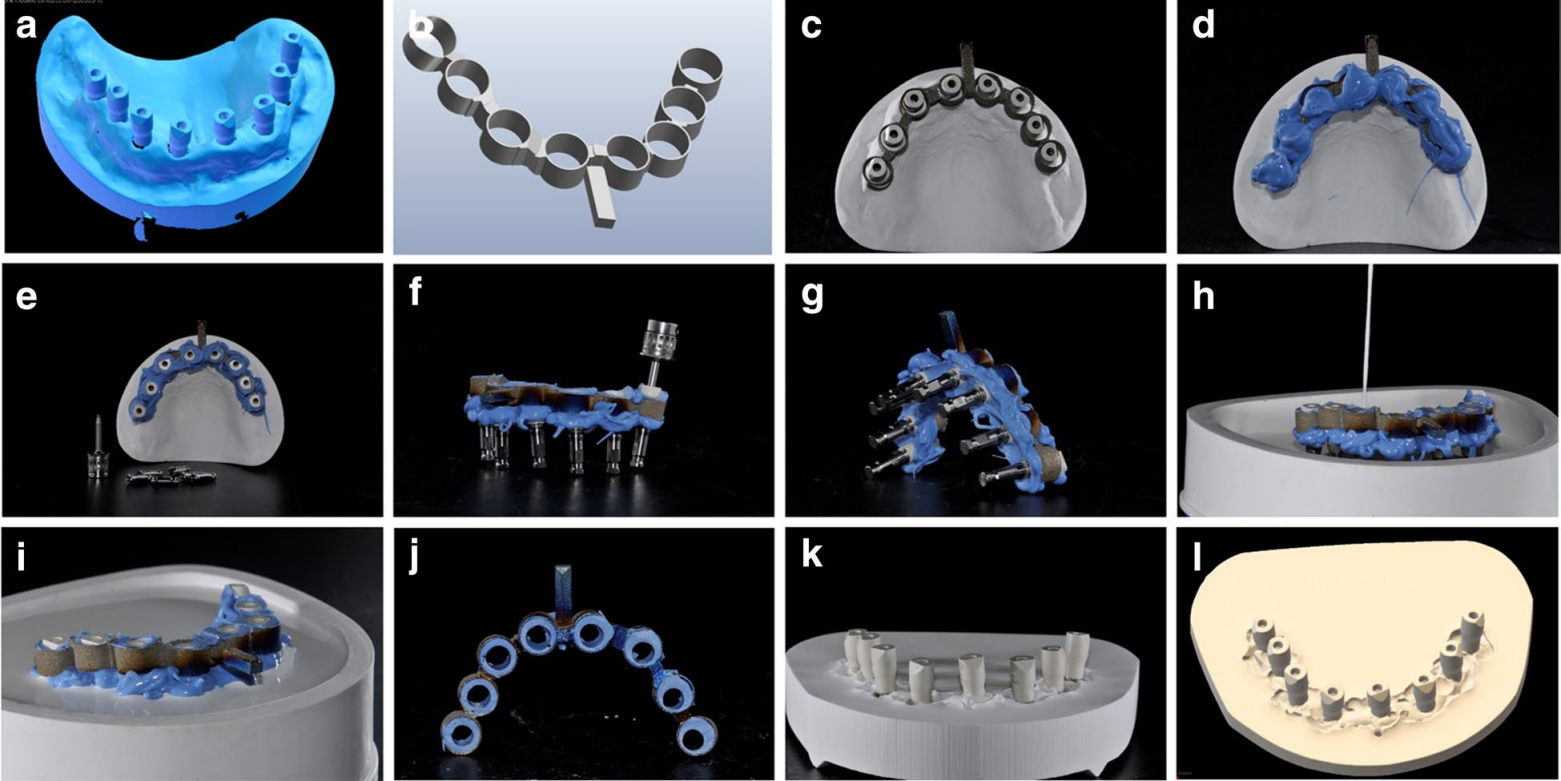
Procedures with the SI. The reference virtual model captured with 7Series® (**a**) was used to design a SI (**b**). The SI was tried on the reference stone cast model (**c**) and therefore used to capture a physical impression (**d**) of the implant position with polyether (Impregum®). After hardening of the material, the SBs head were made free using a blade (**e**), the SBs were unscrewed and the SI was removed with all transfers inside. This SI was then turned and the implant analogs were carefully screwed into the SBs (**f**, **g**). Then the SI was used to pour a plaster cast (**h**, **i**). The implant SBs were not unscrewed and the SI was carefully detached, once again using a blade (**j**). The SI-derived cast (**k**) was scanned with a desktop scanner (D15®; Camcube, Montreal, Canada) (**l**)

The reference gypsum model was then scanned with a powerful certified desktop scanner (7Series®, Dentalwings, Montreal, Canada). In this way, a reference virtual model (RVM) was generated (Fig. 2a), trimmed with the same aforementioned template and saved in a specific folder, in STL format. This reference scan was also sent to a milling centre and used to design a custom-made SI with CAD software (Meshmixer®, Autodesk, San Rafael, CA, USA). The SI was designed with a series of hollow cylinders to embrace the SBs, connected by a bar (Fig. 2b), and fabricated through a titanium laser sintering procedure (TruPrint1000®, Trumpf, Ditzingen, Germany) (Fig. 2c). Then, the SI was used to capture a physical impression of the implant SBs on the reference gypsum model, using a minimum amount of polyether (Impregum®, 3 M ESPE, Maplewood, MN, USA) (Fig. 2d). After hardening of the material (Fig. 2e), the implant SBs were unscrewed and the SI was removed with all transfers inside. This SI was then turned and the implant analogues were carefully screwed into the SBs (Fig. 2f and Fig. 2g); subsequently, this index was used to pour a second SI-derived gypsum model (Fig. 2 h and Fig. 2i). The implant SBs were not unscrewed, and the SI was carefully detached using a blade (Fig. 2j). The SI-derived cast (Fig. 2k) was then scanned with another desktop scanner (D15®; Camcube, Montreal, Canada) (Fig. 2l). This file was trimmed as previously described, and saved in STL format. Finally, all the IOSs-derived files (virtual models from CS 3700® and Emerald S®) and the desktop scan of the SI-derived cast were compared with the RVM, to evaluate trueness.

The overall trueness of the virtual models was assessed through the superimposition of library files, i.e. non-uniform rational basis splines (NURBS), to approximate what occurs in the early phases of the CAD modelling. In detail, this evaluation occurred after replacing, within each virtual model (the RVM from the reference desktop scanner, the SI-derived and IOSs-derived models), the 8 SB meshes with the corresponding SB library file, downloaded from the official library of the manufacturer. A reverse engineering software (Studio 2012®, Geomagic, Morrisville, NC, USA) was used to perform these superimpositions, proceeding first with the manual identification of anatomical landmarks on the SB surfaces (first rough alignment), and then launching a specific robust-iterative-closest-point (RICP) algorithm, capable of automatically overlapping the surfaces of the SBs. Then, a new STL file (which included only 8 SBs library files free in the space, representing the implant positions for each virtual model) was generated and saved. Finally, the new STL files of the implant positions derived from the two IOSs and the SI-derived cast were superimposed on the RVM, for trueness evaluation. The superimposition proceeded using the aforementioned reverse engineering software, once again through the manual identification of specific landmarks on the surface of the SBs (first rough alignment), and then utilising the RICP algorithm, capable of overlapping the surfaces of the models. The parameters for this superimposition were set at a minimum of 100 iterations per case, and the distances between the RVM and the IOS and SI-derived models were minimised using a point-to-plane (PTP) method. The congruence between corresponding structures was calculated, and the software computed the mean ± standard deviation (SD) of the distances (in μm) between the superimposed models. Finally, colourimetric maps were generated for the immediate visualisation of the distances between the models.

**Fig. 3:**
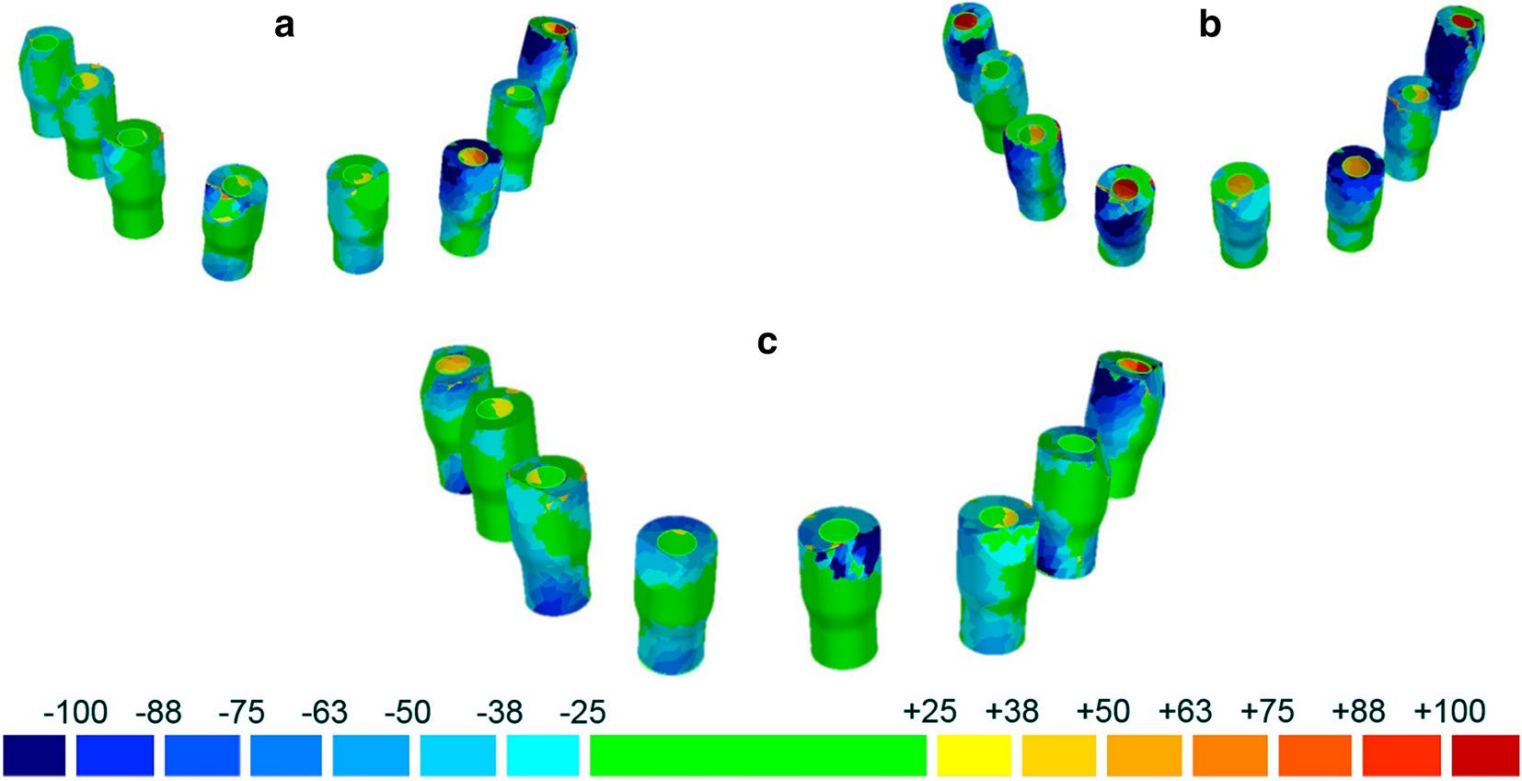
Trueness of the SI-derived and of IOSs-derived models in μm: colorimetric map. In this picture, the best results obtained with each IOS were reported: **a** CS 3700® (28 ± 24 μm); **b** Emerald S® (46 ± 34 μm); **c** SI-derived model scanned with D15® (29 ± 26 μm)

At the end, the most accurate model was the SI-derived with an average trueness of 29 μm (± 26). The distances between the IOS-derived models and the RVM were higher, with an overall mean trueness of 42.4 μm (± 14.7) for CS 3700® and of 52.2 μm (± 4.6) for Emerald S®, respectively (Fig. 3a–c).

## Limitations

In this study, the SI-derived model demonstrated a better mean trueness (29 μm) than the IOS-derived models (42.4 μm for CS 3700® and 52.2 μm for Emerald S®, respectively). This may have important clinical implications, and suggest the use of SI as an ideal solution for capturing an accurate impression, for the design and manufacture of clinically precise implant-supported FAs, in full accordance with the results reported by Schmidt et al. [12] and Mandelli et al. [13]. In only one scan (best mesh with CS 3700®) was the IOS result comparable (28 μm) to that obtained with the SI-derived model. These results demonstrate the meaningful advancements made by IOSs in the last few years, but also how delicate the scanning process can be, as different scans produce different results. Therefore, despite the enormous progress made in the accuracy of the IOSs, difficulties persist in scanning the completely edentulous patient, who needs prosthetic restorations with FA supported by 6 or more fixtures [3–5]. These are determined by the difficulty of the IOS to correctly read the distances between the different SBs, and the spatial and temporal progression of the scan [4–6, 14, 15]. SI also allows for control over the quality of the virtual models derived by the IOSs, using high-quality reference machines (CMM or industrial desktop scanners). This control is not possible using IOSs only. The patient does not perceive the SI as a conventional physical impression, as the quantity of material is limited to a minimum; in the case of implants already parallelised and for impressions on multi-unit-abutments (MUA), polyether can be replaced by plaster, for even higher accuracy in the transfer of the implant position [13].

More studies are needed to validate the clinical use of SIs in the impression of the completely edentulous patient, who needs rehabilitation with an implant-supported FA. In particular, the present study is in vitro and based on a limited number of scans and models. Although the simulation of the clinical situation may be valid, more samples are needed to draw specific conclusions. In addition, the present study used a laboratory desktop scanner to capture the RVM; an industrial optical scanner or, even better, a CMM would certainly have been more appropriate machines for reference measurements. The SI approach has limitations: it is not entirely digital, since it passes through the capture of a physical impression, and therefore requires the casting of a derived model, to be scanned with a desktop scanner. The possibility of manufacturing a customised tray is undoubtedly a huge advantage, as it limits the quantity of material needed, reduces the patient’s discomfort and increases the accuracy of the impression. However, an extra intraoral scan is required for the design and fabrication of the SI, which must be modelled and printed in 3D. This process requires time and skills. Finally, the SI is not able to provide information on the patient’s soft tissues and the SI-derived model must, in any case, be integrated into a sequence of digital acquisitions obtained using IOS [13, 14]. Further studies are needed to obtain more evidence on the use of SI in the impression of the completely edentulous patient who requires implant rehabilitation.

## Data Availability

The.STL files and the 3D surface models obtained in this study belong to the authors, and are therefore available only upon reasonable request, after approval by all the authors.

## Abbreviations

SI: Solid index
FA: Full-arch
IOS: Intraoral scanners
SB: Scanbody
RVM: Reference virtual model
CAD: Computer-aided-design
3D: Three-dimensionally
CMM: Coordinate measuring machine
SIIP: Solid index impression protocol
STL: Stereolithographic
NURBS: Non-uniform rational basis splines
RICP: Robust-iterative-closest-point
PTP: Point-to-plane
MUA: Multi-unit-abutments
